# COVID-19 is rapidly changing: Examining public perceptions and behaviors in response to this evolving pandemic

**DOI:** 10.1371/journal.pone.0235112

**Published:** 2020-06-23

**Authors:** Holly Seale, Anita E. Heywood, Julie Leask, Meru Sheel, Susan Thomas, David N. Durrheim, Katarzyna Bolsewicz, Rajneesh Kaur

**Affiliations:** 1 School of Public Health and Community Medicine, University of New South Wales, Sydney, NSW, Australia; 2 Faculty of Medicine and Health, University of Sydney, Sydney, NSW, Australia; 3 National Centre for Immunisation Research and Surveillance, Kids Research, Sydney Children’s Hospitals Network, Westmead, NSW, Australia; 4 National Centre for Epidemiology and Population Health, Research School of Population Health, ANU College of Health and Medicine, The Australian National University, Acton, ACT, Australia; 5 School of Medicine and Public Health, University of Newcastle, Wallsend, NSW, Australia; 6 Office of Medical Education, Faculty of Medicine, University of New South Wales, Sydney, NSW, Australia; Chinese Academy of Medical Sciences and Peking Union Medical College, CHINA

## Abstract

**Background:**

Since the emergence of SARS-CoV-2, the virus that causes coronavirus disease (COVID-19) in late 2019, communities have been required to rapidly adopt community mitigation strategies rarely used before, or only in limited settings. This study aimed to examine the attitudes and beliefs of Australian adults towards the COVID-19 pandemic, and willingness and capacity to engage with these mitigation measures. In addition, we aimed to explore the psychosocial and demographic factors that are associated with adoption of recommended hygiene-related and avoidance-related behaviors.

**Methods:**

A national cross-sectional online survey of 1420 Australian adults (18 years and older) was undertaken between the 18 and 24 March 2020. The statistical analysis of the data included univariate and multivariate logistic regression analysis.

**Findings:**

The survey of 1420 respondents found 50% (710) of respondents felt COVID-19 would ‘somewhat’ affect their health if infected and 19% perceived their level of risk as high or very high. 84·9% had performed ≥1 of the three recommended hygiene-related behaviors and 93·4% performed ≥1 of six avoidance-related behaviors over the last one month. Adopting avoidance behaviors was associated with trust in government/authorities (aOR: 6.0, 95% CI 2.6–11·0), higher perceived rating of effectiveness of behaviors (aOR: 4·0, 95% CI: 1·8–8·7), higher levels of perceived ability to adopt social distancing strategies (aOR: 5.0, 95% CI: 1·5–9.3), higher trust in government (aOR: 6.0, 95% CI: 2.6–11.0) and higher level of concern if self-isolated (aOR: 1.8, 95% CI: 1.1–3.0).

**Interpretation:**

In the last two months, members of the public have been inundated with messages about hygiene and social (physical) distancing. However, our results indicate that a continued focus on supporting community understanding of the rationale for these strategies, as well as instilling community confidence in their ability to adopt or sustain the recommendations is needed.

## Introduction

In the course of four months, since the first reports about a novel strain of coronavirus, severe acute respiratory syndrome coronavirus 2 (SARS-CoV-2) emerging in December 2019 [1, 2], countries around the world have introduced a range of community mitigation strategies with the aim to lower the trajectory of this pandemic by reducing transmission, and avoid overwhelming health services. Community mitigation strategies refer to measures that people, and communities can take to slow the spread of infection during a period when vaccines and/or medical treatments that are not available [3]. They include the use of personal protective measures for everyday use (e.g., voluntary home isolation of ill persons, respiratory etiquette, and hand hygiene); community measures aimed at increasing social distancing (e.g., maintaining a physical distance of 1·5–2·0 meters between people, staying at home and postponing or cancelling gatherings); and environmental measures (e.g., routinely disinfecting surfaces). In some settings these strategies are voluntary, whereas in others they are now enforced (such as, via fines and/or jail time).

Governments are implementing strategies at large-scale that have previously been used in limited ways and for limited time periods i.e. during Ebola, avian influenza outbreaks and SARS. This means a large proportion of the population do not have prior experience undertaking these strategies. People’s ability to comply with recommendations during emergency situations is influenced by a range of modifiable and nonmodifiable factors including: (1) what people perceive their susceptibility to infection to be [4]; (2) whether they perceive the infection to be serious, if acquired; (3) whether they have the necessary capacity, confidence and resources to comply with the strategies [5]; and (4) their sociodemographic status [6]. Effective control of this pandemic requires an understanding of people’s perceptions about their willingness, motivation and ability/capacity to adopt strategies and how this relates to their perceived risk [7]. Perceived costs, perceptions about the benefits of the behaviors and the perceived impact of an individual's behavior on another’s health will also influence engagement with these behaviors [8, 9].

In Australia, the government has recommended specific behaviors that can be classed as hygiene-related and avoidance related. To engage in these behaviors, people will weigh up the perceived costs and benefits related to themselves and others. It is therefore important to understand community perceptions and behaviors in order to develop effective messages. Accordingly, we carried out a cross sectional online survey of a large, demographically representative sample of the population of Australia in March 2020.

## Materials and methods

### Cross sectional online survey

We conducted an online survey of Australian residents via a market research company (Quality Online Research (QOR)) between 18 and 24 March 2020. This sample size provided us with a sample error of ±3%. Proportional quota sampling was used to ensure that respondents were demographically representative of the general public, with quotas based on age, gender and state/territory. Respondents were required to be 18 years or older and to speak English. Respondents earned points for completing the survey.

### Ethics statement

Ethics approval for the study was obtained from the University of New South Wales HREAP G: Health, Medical, Community and Social (HC200190). After reading the participant information, consent was implied if the person completed the survey and submitted it via the QOR website. No personal identifiers were collected.

### Survey design

The questions for this survey were adapted from published studies by HS during the 2009 influenza H1N1/A pandemic [10, 11]. The study tool is available upon request. Two primary outcome variables used were hygiene-related and avoidance-related behaviors. (see Table 1). Ten items were used to assess respondent perceptions about the COVID-19 pandemic, including perceived risk level and impact on health (if infected). 8/10 items were phrased as statements, with Likert response options scored as 5 for strongly agree through to 1 for strongly disagree. Two items measuring participants level of worry about current Covid-19 were used on a 5 point Likert scale ranging from 1 for strongly disagree to 5 for strongly agree, these were combined and changed into a dichotomous scale of high and low.

**Table 1:** Adoption of hygiene-related and avoidance-related behaviors.in response to COVID-19.

**Hygiene-related behaviors: actions taken over last month due to COVID-19 (Cronbach’s alpha = 0·701)**	**Number (%)**	**Standardised loading**
Increased the time I spent cleaning or disinfecting things I might touch, such as door knobs	537/1420 (37·8)	0.740
Washed my hands with soap and water more often than usual	1088/1420 (76·6)	0.763
Used alcoholic hand gel or hand sanitizer more than usual	806/1420 (56·8)	0.807
**Avoidance-related behaviors: Actions taken over last month due to COVID-19 (Cronbach’s alpha = 0**·**797)**		
Deliberately cancelled or postponed a social event	519/1161* (44·7)	0.814
Cancelled or delayed travelling overseas	407/811* (50·2)	0.745
Reduced my use of public transport	448/880* (50·9)	0.816
Kept away from crowded places generally	889/1332* (66·7)	0.778
Performed ≥ 1 of three recommended behaviors	1205/1420 (84·9)	
Performed ≥1 of six avoidance behaviors	1326/1420 (93·4)	

*Total number of participants lower than overall total of 1420 due to exclusion of participants who ticked ‘not applicable’ options. ^#^ 587 participants had children of school or childcare going age.

Respondents were asked to rate the perceived level of effectiveness of 13 items in reducing the risk from COVID-19 on a 5-point scale These items included those promoted by the government and those that were not (mask use when not symptomatic, taking antibiotics). The strategies were grouped into: (1) hygiene related behaviors (hand washing/sanitizing, cleaning surfaces) and (2) avoidance-related behaviors (avoiding crowds, public transport, and complying with quarantine restrictions). Given the relative novelty of social distancing for the Australian community, we also included a question that assessed the respondent’s ability to adopt 6 different social distancing strategies (working from home, keeping children home from school, avoiding travelling, avoiding large crowds, quarantine if exposed, and isolation if symptomatic) with possible response options scored as 1 for very high and 5 for very low. The last section of the survey included six items focused on self-isolation. Respondents were asked to comment on their willingness to comply, their level of concern regarding the impact on being placed into self-isolation (at home), their ability to comply, their access to assistance from family/friends and issues they have with the strategy. All predictor variables and the items and scales are described in the supplementary materials.

We collected data on gender, age, education and employment status, children (including attendance at childcare/school), country of birth/language spoken at home, whether they identify as Aboriginal and/or Torres Strait Islander, international travel patterns since 1 January 2020, private healthcare insurance coverage, income protection insurance, the presence of any chronic illness and self-reported health status (very good, good, moderate, poor, very poor). Due to the promotion of social distancing and working from home by the Australian Government, we also included two items that assessed access to internet and a computer at home.

#### Analysis

Correlation matrix of all scales was studied. Items of scales with low correlation with the rest of the items of a scale were excluded. This was followed by Principle Component analyses using direct Oblimin rotation to determine the number of components to retain and loading of items for all scales. Cohen alpha was used to check the internal consistency of all items of the scale (See S1 Table). Univariate associations were tested between primary outcome measures and demographic factors. Univariate associations between worry and outcome measures was also assessed using univariate logistic regression. Two separate multivariate logistic regression models were used to measure the associations of perception factors with each outcome factor after adjusting for independent variables. Demographic variables with a P<0.25 in the univariate analysis were used to adjust the models. In Model one hygiene behaviour was tested as the outcome and was adjusted for gender, country of birth, travelled overseas and worry variables. Model two tested avoidance behaviour as the outcome and was adjusted for age, gender, country of birth, employment, current health conditions and worry variables. For all analyses, P values of less than 0.05 were considered statistically significant. Data were analyzed using the SPSS software version 26.0 (SPSS Science, Chicago, IL, USA).

Correlation matrix of hygiene related behaviour items demonstrated a moderate correlation of included three items (rs ranged between 0.34 and 0.405). Overall KMO value of 0.65 was adequate for exploratory factor analysis. Correlation matrix for the avoidance behaviour scale revealed that the third and the last items were outliers and were not related to the other items in the scale (rs = 0. 19 and 0.10 respectively) and were therefore excluded. The correlation of the remaining items showed moderate correlation (rs ranged between 0.33 and 0.39). Overall KMO value was 0.66. Standardised loading of items included in these scales is shown in Table 1.

From the rating of level of effectiveness to reduce risk of COVID, fourth and seventh items of the scale demonstrated a low correlation (0.04 and 0.14) with other items of the scale and were therefore excluded. The remaining items of the scale showed moderate to high correlation (rs between 0.49–0.67). Similarly, second item from the rating of ability to adopt social distancing strategies scale was excluded due to its low correlation with rest of the scale items (rs = 0.16). The remaining items of the scale showed moderate correlation (rs between 0.34–0.55). From the variable ‘rating of level of concern if self-isolated’ third item was excluded due to low correlation with other items of the scale (rs = 0.15). The rest of the items of the scale showed moderate correlation (rs between 0.32–0.44). The factor loading of items of these three scales is shown in S1 Table. Combined items for all variables demonstrated a moderate (Cronbach’s α = 0.733) to high (Crohn’s α = 0.877) internal consistency and were therefore retained.

## Results

Of the 1420 respondents, 740 (52%), were female, 47 (3·3%) identified as Aboriginal and/or Torres Strait Islanders, 830 (58·5%) had private health insurance, 792 (55.8%) had children with 211/792 (26·7%) attended childcare/school. Of the respondents, 37 (%) reported knowing of a COVID-19 case amongst their family or friends. Television news was the primary source of information about COVID-19 (n = 724, 51%), followed by government websites (n = 241,17%) and social media (n = 198, 14%). When asked about the level of trust they had in the information coming from the Government, 667 (47%) stated high to very high.

### Perceptions about susceptibility and severity

Respondents ranked their risk of acquiring COVID-19 as very high (n = 71, 5%); high (n = 198, 14%), intermediate (n = 497, 35%), low (n = 397, 28%) and very low (n = 156, 11%). The remaining 100 respondents reported not knowing what their risk was. When it came to perceived impact on their health, 710 (50%) reported that COVID-19 would ‘somewhat’ affect it, while the remaining respondents reported it as: extremely (n = 170, 12%), seriously (n = 326, 23%), not at all (n = 85, 6%) or don’t know (n = 113, 8%). Fifty percent reported changing their personal perception of risk after reading or hearing information in the media or on social media.

### Perceived effectiveness of social mitigation strategies and ability to adopt

**Fig 1:**
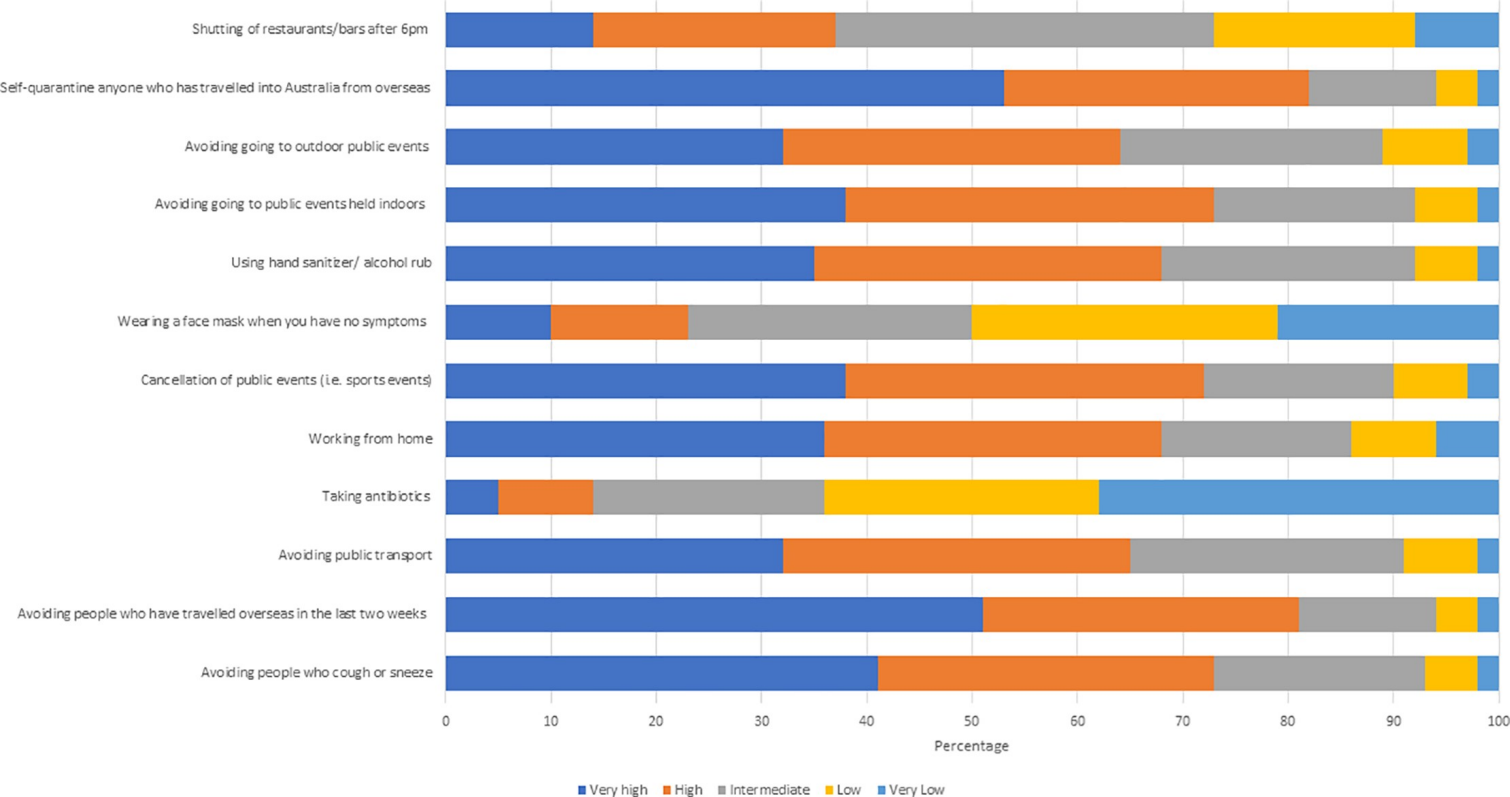
Rating of level of effectiveness of strategies to control Covid-19 outbreak.

Fig 1 shows the perceptions towards the degree of effectiveness of measures to reduce personal risk from COVID-19. Self-quarantine of anyone who has travelled into Australia from overseas was considered to have high to very high effectiveness (n = 1171, 82·5%), followed by avoiding people who have travelled overseas (n = 1155, 81·4%). Whereas, only 525 (37%) thought that shutting the restaurants/bars after 6pm would have a high/very high effect and 696 (49%) stated that wearing a mask (when not symptomatic) would be effective. Taking antibiotics was considered to have low to very low effectiveness by most (n = 908, 64%). Beyond perceived effectiveness, respondents were asked to comment on their ability (self-efficacy) to carry out social distancing strategies, 596 (42%) respondents rated their ability to work from home as high/very high.

### Practice of recommended measures/behavior

The most common hygiene-related behavior adopted was washing hands with soap and water (n = 1087, 76·6%), whereas keeping away from crowded places generally was the most common avoidance behavior (n = 947, 66·7%). Overall, 1205 (84·9%) respondents reported undertaking ≥ 1 of three hygiene-related behaviors and 1326 (93·4%) performed ≥1 of six avoidance-related behaviors (Table 1).

Table 2 shows association between demographic characteristics and reported behaviors during COVID-19 pandemic. Five hundred and five (36·3%) respondents considered the hygiene-related and avoidance-related behaviors as ‘the right thing to do’ as their main motivation to comply. Close to 80% of respondents (n = 1127) who reported being worried about COVID-19 (high-very high) were found to have higher engagement with hygiene-related behaviors (OR 4.2, 95% CI: 3.1–5·8) and avoidance-related behaviors (OR 4.0, 95% CI: 2·6–6.2).

**Table 2:** Association between demographic characteristics and adoption of preventive/avoidance strategies during COVID-19 pandemic.

Variable	No (%) of participants	No (%) using ≥hygiene behaviors	Odds ratio (95% CI)	No (%) using ≥avoidance behavior	Odds ratio (95% CI)
Sex					
Women	740 (52·1)	646 (87·3)	1·5 (1·1–2·1) *	707 (95·5)	0.7(0.5–1)
Men	678 (47·7)	559 (82·4)	Ref	618 (91·2)	Ref
Other^#^	2 (0·1)	0 (0)	-	1 (50)	-
Age group					
18–49	803 (56·5)	685 (85·3)	Ref	739 (92)	Ref
≥ 50	617 (43·5)	520 (84·3)	0·9 (0·7–1·2)	587 (95·1)	1·0 (0.7–1.6)*
Aboriginal and/or Torres					
Strait Islander					
Yes	47 (3·3)	40 (85·1)	1.0 (0·5–2.2)	42 (89·4)	0.8 (0·3–2.1)
No	1373 (96·7)	1165 (84·9)	Ref	1284 (93·5)	Ref
Country of birth					
Australia	1096 (77·2)	919 (83·9)	Ref	1011 (92·2)	Ref
Other	324 (22·8)	286 (88·3)	0·7 (0·5–1·0)	315 (97·2)	3.1 (1·7–6·0)*
Working status					
Not working	591 (41·6)	497 (84·1)	1·2 (0·9–1·6)	567 (95·9)	0.7 (0.5–1.2)
Working full/part time	829 (58·4)	708 (85·4)	Ref	759 (91·6)	Ref
Educational attainment	30 (2·1)				
None	131	27 (90)	Ref	28 (93·3)	Ref
School certificate (year 10)	(9·2)235	108 (92·4)	0·5 (0·2–1·9)	119 (90·8)	0·7 (0·2–2.7)
Leaving certificate (year 12)	(16·5)	197 (83·8)	0·6 (0·2–2·1)	219 (93·2)	1·2 (0·3–4·3)
Trade/apprenticeship/cert	483 (34)	405 (83·9)	0·6 (0·2–2.0)	449 (93)	0·9 (0·3–3·1)
Bachelor’s degree	379 (26·7)	328 (86·5)	0·8 (0·2–2·7)	359(94·7)	2.0 (0·6–6.7)
Masters or higher	162 (11·4)	140 (86·4)	0·8 (0·2–2·7)	152(93·8)	2.6 (0·7–9.4)
Children in household					
Attending childcare/school	212 (14·9)	184 (86·6)	1.2 (0·8–1·9)	195 (92)	1.4 (0·8–2.5)
Not attending childcare/school or no children	1208 (85·1)	1021 (84·5)	Ref	1131(93·6)	Ref
Travelled overseas in 2020	222 (15·6)	201 (90·5)	1.9(1·2–3.1)*	206 (92·8)	2.3 (1.4–3.9*
No	1198 (84·4)	1004(83·8)	Ref	1120 (93·5)	Ref
Have private health	830 (58·5)	712(85·.8)	1.2(0·9–1·7)	(93)	1.2 (0·8–1·9)
Insurance					
No	590 (41·5)	493(83·6)	Ref	554 (93·9)	Ref
Health rating					
Very good/good	1009 (71·1)	861 (8)6·9	Ref	945 (93·7)	Ref
Moderate	294 (20·7)	241 (82)	0·6 (0·4–1.1)	271 (92·2)	1·1 (0·5–2·7)
Poor/very poor	117 (8·2)	95 (81.2)	0·7 (0.5–1.0)	110 (94)	0·8 (0·5–1·3)
Chronic health condition					
Present	363 (25·6)	317 (87·3)	1.5(0·7–3.3)	345 (95)	1·2 (0·4–3.2)
None	1057 (74·4)	888 (84)	Ref	981(92·8)	Ref

*P<0·05

# Not included in OR calculations.

Controlling for demographic and worry variables, there was a higher association between performance of hygiene-related behaviors and trust in government/authorities (aOR: 2·7, 95% CI 1·4–5.1), perceived high severity if infected (aOR: 1·4, 95% CI 1·2–3.0), higher levels of belief in the effectiveness of behaviors (aOR 3·2, 95% CI: 1.4–7.2), higher ability to adopt social distancing strategies (aOR: 3.6,95% CI 1·6–7.0), higher levels of concern if self-isolated (aOR: 2·4 95% CI: 1·1–4·0) and intermediate to higher level of risk perception (aOR: 1.6, 95% CI: 1·1–2·0, aOR: 2.0, 95% CI: 1·2–3.5) led to performance of recommended behaviors. Reporting the use of avoidance behaviors was more likely in respondents who: trusted government/authorities (aOR: 6.0, 95% CI 2.6–11·0), rated effectiveness of behaviors higher (aOR: 4.0, 95% CI: 1.3–12.7), and indicated a higher ability to adopt social distancing strategies (aOR: 5.0, 95% CI 1·5–13.6), perceived high severity if infected (aOR: 1.8, 95% CI: 1.1–3.0) (Table 3).

**Table 3:** Logistic regression models testing association between perception variables and adoption of hygiene/avoidance strategies during COVID-19 outbreak.

		Association with carrying or ≥1 preventive behavior		Association with carrying out ≥1 avoidance behavior	
Variables	No. (%)	OR (95% CI)	Adjusted OR (95% CI)	OR (95% CI)	Adjusted OR (95% CI)
Trust in government/authorities					
High	1315/1401 (93·9)	4.2 (2·6–6·7)*	2·7 (1·4–5.1)*	5.8 (3.2–10.7)*	6·0 (2.6–11.0)*
Low	86/1401 (6.1)	Ref	Ref	Ref	Ref
Perceived Severity					
High	1219/1400 (85·8)	2·6 (1·8–3·7)*	1·4 (1·1–2·3)*	2·0 (1·2–3·3)*	1·5 (0·7–3.2)
Low	181/1400 (12.7)	Ref	Ref	Ref	Ref
Rating of level of effectiveness of behaviors~					
High	1150 (81)	8.8 (5.0–15.5)*	3·2 (1.4–7.2)*	14.3 (6.0–24.3)*	4·0 (1.88.7)*
Low	270 (19)	Ref	Ref	Ref	Ref
Ability to adopt social distancing strategies					
High	740/1293 (52.1)	5.7 (3.2–10.4)*	3.6 (1.6–7.0)*	15.7 (8.625·8)*	5.0 (1.5–9.3)*
Low	553/1293 (38.9)	Ref	Ref	Ref	Ref
Level of concern if self-isolated^€^					
High	274/1359 (19·3)	1.4 (1·1–2.0)*	2·4 (1·1–4·0)*	1·5 (0·8–2·9	1·8 (1.1–3·0)*
Low	1085/1359 (76.4)	Ref	Ref	Ref	Ref
Level of Risk					
Very low/low	553/1320 (41·9)	Ref	Ref	Ref	Ref
Intermediate	496 /1320 (37·6)	2·0 (1·4–2·8)*	1·6 (1·1–2·0)*	1·5 (1.0–2·4)	1.1 (0·6–1·7)
Very high/high	271/1320 (20·5)	3·1 (1·9–4·9)*	2·0 (1·2–3·5)*	3.0 (1·7–5.4)	1·7 (0·8–3.4)
Impact on health					
No/somewhat	806/1304 (61·8)	Ref	Ref	Ref	Ref
Serious/Extreme	498/1304 (38·2)	1·6 (1·2–2·3)*	1·0 (0·6–1·5)	3.2 (2.0–5.2)*	1·6 (0·9–2.9)

*P<0.05, Two logistic regression models: Model one: Hygiene related behaviour as the outcome, model adjusted for gender, country of birth, travelled overseas, private health insurance, rating of current health and worry, variables with P<0.25 in the univariate analysis. Model two: avoidance behaviours as the outcome, model adjusted for gender, country of birth, employment, ATSI status, travelled overseas, children attending childcare/school or not, and worry, variables with P<0.25.

Six questions focused on self-isolation as a strategy. The majority (n = 1349, 95%) agreed that they could self-isolate if necessary and that they had a family member or friend who could assist them in the event of isolation (n = 1178, 83%). However, respondents did have concerns (high/very high) about not being able to access shops for food/supplies (n = 681, 48%) and not being able to access a primary care provider (n = 553, 39%). Amongst those who felt they could not manage self-isolation at home (n = 122, 8%), the main concerns were centered around carers responsibilities for children, elderly parents and disabled family members.

## Discussion

Our results suggest that a large proportion of respondents have adopted one or more of either the hygiene-related and/or avoidance-related behaviors that had been recommended by the Australian Government. Considering the intense media coverage and government information, it is not surprising that there was a gravitation towards actual (or willingness for) adoption of hygiene strategies including hand washing/sanitizing. While anxiety levels were moderate, concerns were raised about accessing food and medical supplies if placed into self-isolation. Given the limited community behavioral COVID-19 studies published, we have compared our findings to two ongoing community surveys. Firstly, results from an online poll of 14,000 respondents from 14 countries (conducted at the same time point as our study), reported that their respondents in 8/14 countries expressed a belief that social distancing measures such as travel bans, and self-isolation would not prevent the spread of the virus, including participants from Australia (52%) [12]. Secondly, a German survey (conducted a week earlier then our study) identified that respondents had high levels of knowledge, but adoption of important protection behaviors was very low, and risk perceptions were especially low among the elderly [13]. Lastly, a survey from Malaysia reported that a high proportion of respondents were already adopting precautions such as avoiding crowds (83.4%) and practicing proper hand hygiene (87.8%) at the time of their study in late March. However, the wearing of face masks was less common (51.2%) [14].

Amongst our participants, perceived susceptibility to COVID-19 was at an intermediate level. This aligns with results found in a similar study conducted in the UK, which reported that just under half of their cohort (n = 2,108) were likely to acquire COVID-19, while 56% felt that it would have a moderate impact (i.e. would require them to self-care and rest in bed) [15]. In addition, a separate survey of Australian residents also found that two thirds of respondents were at least moderately worried about a widespread COVID-19 outbreak in Australia in early March [16]. Risk perceptions were also found to be moderate (median 5 out of 10) based on an online poll of US residents undertaken in early February [17]. There may be several factors at play here that account for the perceived level of risk. Firstly, people may be unaware of true risk since little reporting has focused on attack rates during the time of the study. Secondly, people may be subject to optimism bias–a phenomenon where people downplay their own risk of an outcome [18]. Thirdly, it is likely that some people assess their risk as being low due to already factoring in a change towards anticipated or already accomplished protective behavior [19]. During events that people deem ‘familiar’, we often see unrealistic optimism because the risk is perceived to be under control, as was the case in 2009 with the influenza pandemic, when adoption of precautions was low and there was a sense of personal security [4]. However, COVID-19 presents as an unfamiliar risk (for the large majority of the population had not experienced outbreaks of SARS or MERS) making the risk less tolerable for those who perceive the situation as uncontrollable [20]. When it comes to perceptions of risk, there are numerous studies documenting how they are associated with the uptake of preventive and/or avoidant behaviors. Studies conducted during/after the 2003 SARS outbreak reported that higher levels of perceived risk/susceptibility of SARS was associated with the adoption of preventive behaviors and also avoidance behaviors [21–24].

While understanding a person’s perception of risk is important, it is not the only condition needed for engagement. Higher risk perceptions may only predict protective behavior when people believe that effective protective actions are available (response efficacy) and when they are confident that they can engage in such protective actions (self-efficacy) [25]. According to Bandura social cognitive theory, an individual’s self-efficacy plays a crucial role on the individual’s likelihood to engage in a desired behavior. If an individual does not believe that he/she can carry out the behavior (i.e. physically distance themselves), there is little motivation to engage [26]. Three-quarters of the study respondents agreed that they could adopt the avoidance-related strategies, with lower scores for working from home and self-isolation at home. When asked whether they had adopted any of the hygiene related strategies, washing or sanitizing hands were the most common responses. These findings have also been replicated in an online survey of 5974 residents from the US and UK, that found that 92% of the cohort would adopt hygiene related behaviors [27]. These represent more readily adoptable strategies, as people in the community understand how to engage in them, believe that the strategy will protect them, and usually have the resources to carry them out. These easy to adopt actions have also been a focus of government mass media messages.

When it came to avoidance behaviors, our respondents were less inclined to rate them as being effective or to have adopted them, in comparison to the preventive behaviors listed above. Perceptions regarding the efficacy of the strategy (as opposed to self-efficacy) have also been found to impact on intentions/likelihood to adopt or actual uptake [23, 28]. It is not surprising that some strategies including social distancing, scored low as people may not understand what the strategy entails, the rationale for its use, or what impact it may have on one’s health. It should also be noted that individuals may not have the capacity or resources to comply with physical distancing measures because they: (1) have extended families living in their households; (2) they have a responsibility to provide care for someone outside of their home; (3) they may reside in share accommodation; (4) may not have access to internet/computer in the home setting or (5) because of the type of job they have they cannot simply shift to working from home. In these settings, providing mass media education is not going to suffice. What is needed is pragmatic solutions that support people financially and socially to participate. Examples including increases in social support and charities to assist with delivery of groceries and meals, home delivery from chemists, telehealth consultations bulk billed, drive through vaccine clinics etc.

It has been suggested that we start to prime people about what additional strategies may still need to be introduced [29]. This would entail talking with them about why the strategy would be implemented; the end-goal of implementing it; what could be the potential impacts; how members of the public engage; and the criteria for its de-escalation. In order to promote cooperation with social (physical distancing) strategies, governments may need to use realistic portrayals (community stories) and role modelling by influential actors in social networks. Observing competent role models perform actions that result in success conveys information to observers about the sequence of actions to use to be successful [30]. Motivation may be helped by creating media campaigns that foster awareness of the recommended behaviors and encourage people to share their strategies for complying with self-isolation and working from home.

Amongst our respondents, older age was associated with the adoption of precautionary behaviors, which aligns with the findings from Singapore and Hong Kong during the 2003 SARS outbreak [7, 23] and some studies during the 2009 H1N1/A pandemic [31, 32]. However, the pattern of age is not straight forward. In contrast to the above studies, others have reported higher levels of adoption of preventive behaviors amongst younger people (18–24 years) in the context of the 2009 influenza pandemic. When it comes to gender, we found that females were more likely to report uptake of both preventive and avoidance behaviors, consistent with studies during SARS and H1N1 pandemic influenza [4, 7, 23, 33]. Earlier studies have indicated that women are more likely to perceive themselves to be susceptible and hence adopt the behaviors [21, 34]. When it comes to country of birth, we found that people born outside of Australia were less likely to adopt behaviors. This finding may relate to the capacity to access information, which at the time, was being disseminated in English and largely through mainstream media conferences and health department websites rather than community and language groups. Further work is needed to explore the associations between country of birth and pandemic-related behaviors.

When asked what would motivate respondents to comply with a social distancing strategy, they nominated ‘I believe it is the right thing to do’ as the primary response. While this answer did not have any significant relationship with the outcome measures reviewed, it is still relevant when it comes to planning communication messages. It suggests that respondents may be influenced by a desire for social approval from others, an idea linked to the model of moral motivation. The model, developed by Brekke et al. (2003), assumes that individuals have preferences for achieving and maintaining a self-image as a socially responsible person [35]. In Brekke et al.'s model, self-image improves when the individual's actual behavior gets closer to her/his view of the “morally ideal” behavior (i.e. the behavior that would maximize social welfare if chosen by every member of society). However, individual’s participation can be conditional on whether they think others are also contributing [36]. Mass media campaigns that frame their messages around a social collective action/power or the inclusion of the general public within a team to assist the community response may be effective. The promotion of prosocial behaviors has been shown to be effective in vaccination uptake and could be adapted in promoting COVID-19 mitigation behaviors, such as how one’s actions can contribute to protecting their grandparents [37]. This idea has been picked up by celebrities and the wider community on twitter under the #LockDownForLove, with people nominating who they are social distancing for. Whether these strategies work, needs to be further examined.

Early results from a study conducted across the US, UK and Germany has suggested that inducing empathy for those most vulnerable to the virus promotes the motivation to adhere to physical distancing [38]. The use of empathy in messaging is not a new concept and has been applied in a range of ways from the promotion of testing/treatment for STIs, through to increasing our acceptance of robots [39, 40]. Empathy is a skill which enables understanding of another person’s experience. Here, people could be asked to imagine the perceptions, needs and impact (health, financial, social) of pandemic COVID-19 amongst our family members/friends.

Our study includes a large, representative cross-section of the adult Australian population. People who could not communicate in English were excluded from the sample, which may have affected representation of ethnic minorities. We also had under-representation of Aboriginal Australians and Torres Strait Islanders and from those in remote settings. Secondly, as participation in our study was on a voluntary basis, this study has potential for self-selection bias by community members who are particularly concerned about this pandemic. We relied on self-reports of behaviors which may have led to over-reported (social desirability bias) However, this may have minimized as the survey was self-complete and anonymous. Lastly, we did not collect information about income level and so unable to comment on variations in behaviors by income.

Based on the available data, it appears that older individuals (aged >60 years) and people with chronic underlying health conditions are particularly susceptible to severe disease. This presents a challenging situation. In the media there is reporting that ‘COVID-19 is causing mild illness’ in the majority but it’s in the best interest of the country to stay home in order to ‘flatten the curve’. This will cause two responses–those who continue with their normal practice (not adopting or complying with the recommendations around social distancing/mitigation strategies) as identified in a proportion of our respondents. Motivating this group (especially those less likely to be at risk or suffer the health impact of COVID-19) to adopt behaviors that require marked change in their routines, beyond those related to personal hygiene. It may prove difficult unless people understand the required behavior, the rationale for it, are given clear and sufficient information about how to comply, and they believe the strategy will have an impact and are motivated to act [9, 23, 41]. They also need to have capacity and opportunity to comply with new behaviors for another 4 to 6 months. In order to engage a community, they need to feel like they are a valued part of a team, and that their contributions are valued and key to the response. Lastly, it is essential that governments ensure that resources, legalization and support measures are in place in order to facilitate community participation in community mitigation strategies.

## Supporting information

S1 Table List of original and recoded predictor variables. (DOCX)

## References

[1] Anastassopoulou C, Russo L, Tsakris A, Siettos C. Data-based analysis, modelling and forecasting of the COVID-19 outbreak. PloS one. 2020;15:e0230405 10.1371/journal.pone.0230405 32231374 PMC7108749

[2] Guan WJ, ZY, Hu Y, Liang WH, Ou CQ, He JX, et al Clinical Characteristics of Coronavirus Disease 2019 in China. N Engl J Med. 2020;382:1708–20. 10.1056/NEJMoa2002032 32109013 PMC7092819

[3] Anderson RM, Heesterbeek H, Klinkenberg D, Hollingsworth TD. How will country-based mitigation measures influence the course of the COVID-19 epidemic? The Lancet. 2020;395:931–4. 10.1016/S0140-6736(20)30567-5 PMC7158572 32164834

[4] Rubin GJ, Amlôt R, Page L, Wessely S. Public perceptions, anxiety, and behaviour change in relation to the swine flu outbreak: cross sectional telephone survey. Br Med J. 2009;339:b2651. 19574308 10.1136/bmj.b2651 PMC2714687

[5] Lau JT, Griffiths S, Choi KC, Lin C. Prevalence of preventive behaviors and associated factors during early phase of the H1N1 influenza epidemic. Am J Infect Control. 2010;38(5):374–80. Epub 2010/06/24. 10.1016/j.ajic.2010.03.002 . 20569849 PMC7132693

[6] Blake KD, Blendon RJ, Viswanath K. Employment and compliance with pandemic influenza mitigation recommendations. Emerging infectious diseases. 2010;16(2):212–8. 10.3201/eid1602.090638 . 20113549 PMC2958001

[7] Leung GM, Lam TH, Ho LM, Ho SY, Chan BHY, Wong IOL, et al The impact of community psychological responses on outbreak control for severe acute respiratory syndrome in Hong Kong. Journal of epidemiology and community health. 2003;57:857 10.1136/jech.57.11.857 14600110 PMC1732323

[8] Hershey JC, Asch DA, Thumasathit T, Meszaros J, Waters VV. The Roles of Altruism, Free Riding, and Bandwagoning in Vaccination Decisions. Organizational Behavior and Human Decision Processes. 1994;59(2):177–87. 10.1006/obhd.1994.1055.

[9] Bish A, Michie S. Demographic and attitudinal determinants of protective behaviours during a pandemic: a review. British journal of health psychology. 2010;15:797–824. 10.1348/135910710X485826 20109274 PMC7185452

[10] Seale H, McLaws M-L, Heywood AE, Ward KF, Lowbridge CP, Van D, et al The community's attitude towards swine flu and pandemic influenza. Medical Journal of Australia. 2009;191:267–9. 19740048 10.5694/j.1326-5377.2009.tb02781.x

[11] Seale H, Heywood A, McLaws M, Ward K, Lowbridge C, Van D, et al Why do I need it? I am not at risk! Public perceptions towards the pandemic (H1N1) 2009 vaccine. BMC Infectious Diseases. 2010;10(99):1–9. 20403201 10.1186/1471-2334-10-99 PMC2864274

[12] Public divided on whether isolation, travel bans prevent COVID-19 spread; border closures become more acceptable–Ipsos survey [Internet]. Toronto, Canada 2020

[13] Betsch C. How behavioural science data helps mitigate the COVID-19 crisis. Nature Human Behaviour. 2020. 10.1038/s41562-020-0866-1 PMC7101898 32221514

[14] Azlan AA, Hamzah MR, Sern TJ, Ayub SH, Mohamad E. Public knowledge, attitudes and practices towards COVID-19: A cross-sectional study in Malaysia. PloS one. 2020;15(5):e0233668 10.1371/journal.pone.0233668 32437434 PMC7241824

[15] Atchison CJ, Bowman L, Vrinten C, Redd R, Pristera P, Eaton JW, et al Perceptions and behavioural responses of the general public during the COVID-19 pandemic: A cross-sectional survey of UK Adults. medRxiv. 2020:2020.04.01.20050039. 10.1136/bmjopen-2020-043577 PMC7783373 33397669

[16] Faasse K, Newby JM. Public perceptions of COVID-19 in Australia: perceived risk, knowledge, health-protective behaviours, and vaccine intentions. medRxiv. 2020:2020.04.25.20079996. 10.1101/2020.04.25.20079996 PMC7561403 33117223

[17] McFadden SM, Malik AA, Aguolu OG, Willebrand KS, Omer SB. Perceptions of the adult US population regarding the novel coronavirus outbreak. PloS one. 2020;15(4):e0231808 10.1371/journal.pone.0231808 32302370 PMC7164638

[18] Sharot T. The optimism bias. Current biology: CB. 2011;21:R941–5. 10.1016/j.cub.2011.10.030 . 22153158

[19] Brewer NT, Chapman GB, Gibbons FX, Gerrard M, McCaul KD, Weinstein ND. Meta-analysis of the relationship between risk perception and health behavior: the example of vaccination. Health Psychol. 2007;26:136–45. 10.1037/0278-6133.26.2.136 . 17385964

[20] Brug J, Aro AR, Richardus JH. Risk Perceptions and Behaviour: Towards Pandemic Control of Emerging Infectious Diseases. International Journal of Behavioral Medicine. 2009;16(1):3 10.1007/s12529-008-9000-x 19127440 PMC7090788

[21] Brug J, Aro AR, Oenema A, de Zwart O, Richardus JH, Bishop GD. SARS risk perception, knowledge, precautions, and information sources, the Netherlands. Emerg Infect Dis. 2004;10:1486–9. 10.3201/eid1008.040283 15496256 PMC3320399

[22] Jones JH, Salathé M. Early Assessment of Anxiety and Behavioral Response to Novel Swine-Origin Influenza A(H1N1). PloS one. 2009;4:e8032 10.1371/journal.pone.0008032 19997505 PMC2779851

[23] Lau JTF, Yang X, Tsui H, Kim JH. Monitoring community responses to the SARS epidemic in Hong Kong: from day 10 to day 62. Journal of epidemiology and community health. 2003;57:864 10.1136/jech.57.11.864 14600111 PMC1732318

[24] Blendon RJ, Benson JM, DesRoches CM, Raleigh E, Taylor-Clark K. The public's response to severe acute respiratory syndrome in Toronto and the United States. Clin Infect Dis. 2004;38(7):925–31. Epub 2004/03/23. 10.1086/382355 . 15034821

[25] Rimal RN, Real K. Perceived Risk and Efficacy Beliefs as Motivators of Change. Human Communication Research. 2003;29:370–99.

[26] Bandura A. Social Cognitive Theory and Exercise of Control over HIV Infection In: DiClemente RJ, Peterson JL, editors. Preventing AIDS: Theories and Methods of Behavioral Interventions. Boston, MA: Springer US; 1994 p. 25–59.

[27] Geldsetzer P. Use of Rapid Online Surveys to Assess People's Perceptions During Infectious Disease Outbreaks: A Cross-sectional Survey on COVID-19. J Med Internet Res. 2020;22:e18790 10.2196/18790 32240094 PMC7124956

[28] Lau JTF, Yang X, Tsui H, Pang E, Kim JH. SARS preventive and risk behaviours of Hong Kong air travellers. Epidemiology and infection. 2004;132:727–36. 10.1017/s0950268804002225 . 15310175 PMC2870154

[29] Leask J. Leaders can still build our trust to fight the virus—here's how. Sydney Morning Herald. 26 3 2020.

[30] Schunk DH. Social cognitive theory and self-regulated learning In: Zimmerman BJ, Schunk DH, editors. Self-regulated learning and academic achievement: Theoretical perspectives. Mahwah, NJ Erlbaum; 2001 p. 125–51

[31] Marshall H, Ryan P, Roberton D, Street J, Watson M. Pandemic influenza and community preparedness. American journal of public health. 2009;99 Suppl 2(2):S365–S71. 10.2105/AJPH.2008.153056 . 19797751 PMC4504368

[32] Cowling BJ, Ng DM, Ip DK, Liao Q, Lam WW, Wu JT, et al Community psychological and behavioral responses through the first wave of the 2009 influenza A(H1N1) pandemic in Hong Kong. J Infect Dis. 2010;202:867–76. 10.1086/655811 . 20677945

[33] Eastwood K, Durrheim D, Francis JL, d'Espaignet ET, Duncan S, Islam F, et al Knowledge about pandemic influenza and compliance with containment measures among Australians. Bulletin of the World Health Organization. 2009;87:588–94. 10.2471/blt.08.060772 . 19705008 PMC2733278

[34] Bish A, Michie S. Demographic and attitudinal determinants of protective behaviours during a pandemic: A review. British journal of health psychology. 2010;15:797–824. 10.1348/135910710X485826 20109274 PMC7185452

[35] Brekke KA, Kverndokk S, Nyborg K. An economic model of moral motivation. Journal of Public Economics. 2003;87:1967–83.

[36] Bardsley N, Sausgruber R. Conformity and reciprocity in public good provision. Journal of Economic Psychology. 2005;26:664–81.

[37] Brewer NT, Chapman GB, Rothman AJ, Leask J, Kempe A. Increasing Vaccination: Putting Psychological Science Into Action. Psychological Science in the Public Interest. 2017;18:149–207. 10.1177/1529100618760521 29611455

[38] Pfattheicher S, Nockur L, Böhm R, Sassenrath C, Petersen M. The emotional path to action: Empathy promotes physical distancing during the COVID-19 pandemic. 2020 10.1177/0956797620964422 32993455

[39] Grow JM, Christopher SA. Breaking the Silence Surrounding Hepatitis C by Promoting Self-Efficacy: Hepatitis C Public Service Announcements. Qualitative Health Research. 2008;18:1401–12. 10.1177/1049732308322603 18832769

[40] Pereira A, Leite I, Mascarenhas S, Martinho C, Paiva A, editors. Using Empathy to Improve Human-Robot Relationships. Human-Robot Personal Relationships; 2011; Berlin, Heidelberg.

[41] Tang CS, Wong CY. An outbreak of the severe acute respiratory syndrome: predictors of health behaviors and effect of community prevention measures in Hong Kong, China. Am J Public Health. 2003;93:1887–8. 10.2105/ajph.93.11.1887 14600058 PMC1448068

