# Supplementary material for: COVID-19 is rapidly changing: Examining public perceptions and behaviors in response to this evolving pandemic
Source: PLoS One. 2020 Jun 23;15(6):e0235112. doi: 10.1371/journal.pone.0235112 (PMC7310732; doi:10.1371/journal.pone.0235112)
Supplement: S1 Table — (DOCX) [file pone.0235112.s001.docx]

**Supplementary materials**

**List of original and recoded predictor variables.**

| Rating of level of effectiveness to reduce risk of COVID-19 13 items on a 5 point Likert type scale ranging from 1= Very Low 3=Intermediate to 5= Very High, recoded into dichotomous High and Low. (Cronhnbach’s alpha=0.872) | Mean | SD | Standardised factor loadings |
| --- | --- | --- | --- |
| 1. Avoiding people who cough or sneeze | 4.04 | 1.00 | 0.720 |
| 1. Avoiding people who have travelled overseas in the last two weeks | 4.25 | 0.95 | 0.738 |
| 1. Avoiding public transport | 3.85 | 1.03 | 0.731 |
| 1. Working from home | 3.83 | 1.18 | 0.638 |
| 1. Cancellation of public events (i.e. sports events) | 3.98 | 1.04 | 0.789 |
| 1. Using hand sanitiser/ alcohol rub | 3.94 | 0.99 | 0.626 |
| 1. Avoiding going to public events held indoors | 4.02 | 0.99 | 0.808 |
| 1. Avoiding going to outdoor public events | 3.81 | 1.07 | 0.789 |
| 1. Self-quarantine anyone who has travelled into Australia from overseas | 4.29 | 0.93 | 0.733 |
| 1. Shutting of restaurants/bars after 6pm | 3.15 | 1.12 | 0.605 |
| 1. Increased use of household products | 3.08 | 1.14 | 0.486 |
| Trust in government/authorities consists of two items on a 5-point Likert scale ranging from 1= Strongly disagree 3=Unsure to 5= Strongly agree, recoded into dichotomous High and Low. |  |  |  |
| 1. The government made the correct decision to ban large community events (i.e. sports games or concerts) |  |  |  |
| 2. In general, I think the authorities are doing a good job of dealing with the COVID-19 situation. |  |  |  |
| Rating of ability to adopt social distancing strategies consists of six items on a 5-point Likert scale ranging from 1= Very low 3=Medium to 5= Very high, recoded into dichotomous High and Low.  (Crohnbach’s alpha=0.739) |  |  |  |
| 1.Work from home | 2.46 | 2.08 | 0.404 |
| 3. Stay in quarantine at your home (until you get your test results back). | 3.36 | 2.06 | 0.728 |
| 4. Avoid travelling overseas. | 3.44 | 2.14 | 0.605 |
| 5. Avoid going to a large event like a sports match. | 3.83 | 1.73 | 0.639 |
| 6. Self-isolation in one room in your house (because you have the infection). | 3.17 | 1.80 | 0.480 |
| Rating of level of concern if self-isolated consists of six items on a 5-point Likert scale ranging from 1= Strongly disagree 3=Unsure to 5= Strongly agree, recoded into dichotomous High and Low. (Crohnbach’s alpha=0.710) |  |  |  |
| 1. Not being able to access your GP | 3.04 | 1.35 | 0.583 |
| 1. Not being able to attend work | 1.90 | 1.80 | 0.678 |
|  |  |  |  |
| 1. Not being able to go to the shops for food or other supplies | 3.28 | 1.35 | 0.703 |
| 1. Not being able to have physical contact with your family or friends | 2.96 | 1.41 | 0.759 |
| 1. Not being able to go out to events/sports games. | 1.64 | 1.43 | 0.707 |
| Level of risk of catching COVID-19 during this pandemic measured on a 5 point Likert scale ranging from 1= Very Low 3=Intermediate to 5= Very High. Recoded into three categories: 1= Very low/low 2=Intermediate 3= Very high/high |  |  |  |
| Impact on health measured on a 5 point Likert scale ranging from 1=Not at all 3=don’t know 5=Extremely. |  |  |  |
